# Feasibility study on a longer side-alternating vibration therapy protocol (15 min per session) in children and adolescents with mild cerebral palsy

**DOI:** 10.3389/fped.2023.1231068

**Published:** 2023-08-15

**Authors:** Alena Adaikina, José G. B. Derraik, Janene McMillan, Patricia Colle, Paul L. Hofman, Silmara Gusso

**Affiliations:** ^1^ Liggins Institute, University of Auckland, Auckland, New Zealand; ^2^ Department of Paediatrics: Child and Youth Health, School of Medicine, Faculty of Medical and Health Sciences, University of Auckland, Auckland, New Zealand; ^3^ Environmental-Occupational Health Sciences and Non-Communicable Diseases Research Group, Research Institute for Health Sciences, Chiang Mai University, Chiang Mai, Thailand; ^4^ Department of Women's And Children's Health, Uppsala University, Uppsala, Sweden; ^5^ Exercise Sciences Department, University of Auckland, Auckland, New Zealand

**Keywords:** 6 min walk test, body composition, cerebral palsy, physical function, protocol duration, side-alternating vibration therapy

## Abstract

**Objective:**

Previous studies on side-alternating vibration therapy (sVT) have usually used a 9 min intervention protocol. We performed a feasibility study aimed at assessing the safety, acceptability, and potential effectiveness of a longer sVT protocol (15 min per session) in children and adolescents with cerebral palsy (CP).

**Methods:**

Fifteen participants aged 5.2–17.4 years (median = 12.4 years) with CP GMFCS level II underwent 20 weeks of sVT consisting of 15 min sessions 4 days/week. Participants were assessed at baseline and after the intervention period, including mobility (six-minute walk-test; 6MWT), body composition (whole-body dual-energy x-ray absorptiometry scans), and muscle function (force plate).

**Results:**

Adherence level to the 15 min VT protocol was 83% on average. There were no adverse events reported. After 20 weeks, there was some evidence for an increase in the walking distance covered in 6MWT (+43 m; *p* = 0.0018) and spine bone mineral density (+0.032 g/cm^2^; *p* = 0.012) compared to baseline.

**Conclusions:**

The 15 min sVT protocol is feasible and well tolerated. The results also suggest potential benefits of this protocol to mobility and bone health. Randomized controlled trials are needed to reliably ascertain the potential effectiveness of a longer sVT protocol on physical function and body composition in young people with CP.

## 1. Introduction

Cerebral palsy (CP) is the most common cause of physical disability in children that presents at birth or in early infancy as a result of non-progressive encephalopathies of varied aetiology (1). Clinical symptoms may include impaired muscle function, reduced bone mineral density (BMD) (2), reduced muscle mass (3), as well as varying degrees of mobility impairments (4) and reduced walking endurance (5). The management of CP involves a multidisciplinary approach that includes therapeutic, pharmaceutical, and surgical interventions aimed at increasing mobility, improving muscle and bone health, thereby promoting overall health and well-being (1, 6).

Over the last two decades, side-alternating vibration therapy (sVT) has been investigated as an additional tool in the management of CP. The impulses produced by a vibration platform are thought to stimulate muscle spindles, inducing cyclic elongation and contraction of the stimulated muscles (7). sVT has been shown to improve BMD, muscle mass, walking endurance, muscle strength, and coordination in children and adults with CP (8–10). Nonetheless, interpretation of research findings is difficult due to differences in protocols, including frequency, duration of individual sessions, and duration of the intervention (8).

Studies in children and adolescents with CP tend to use a 9 min protocol with three 3 min sVT bouts, separated by 3 min breaks, following the manufacturer's recommendations and previous published studies (10–13). To the best of our knowledge, only one study has evaluated a longer 18 min protocol with six 3 min sVT bouts (14). However, in that study, each sVT bout varied in frequency, foot placement, and participant position on the vibration plate, which differed from the more standardized protocols typically used in studies of sVT in children (10–13). While that study may provide valuable insights, it is difficult to compare their findings to studies using standardized 9 min protocols. In addition, the authors did not provide information on safety (i.e., adverse effects) or acceptability (i.e., adherence to the sVT protocol, reasons for missing sessions or withdrawal) (14). Therefore, it is still unknown whether longer sVT sessions are feasible in children and adolescents with CP or would offer additional health benefits similar to those seen with an increased load in strength training (15). We hypothesized that prolonged exposure to sVT may have a more marked impact on mobility, muscle, and bone health due to the increased stress on the musculoskeletal system, resulting from greater training levels due a higher number of repeated cycles of muscle contraction and relaxation. Therefore, this feasibility study aimed to assess the safety, acceptability, and potential effectiveness of a longer sVT protocol (15 min per session) in children and adolescents with CP.

## 2. Materials and methods

### 2.1. Design

This study was a prospective single-group clinical study with participants assessed before and after completion of 20 weeks of sVT.

### 2.2. Ethics approval

Ethics approval was granted by the Northern X Regional Ethics Committee (NTX/11/05/042 and NTX/11/05/042/AM04). This study followed the principles of the Declaration of Helsinki, adhering to all appropriate guidelines and regulations for medical research (16). Written informed consent was obtained from parents or legal guardians, as well as informed consent (written or verbal) from all able participants. The trial was registered with the Australian New Zealand Clinical Trials Registry (ACTRN12615000092594) on the 5th of May 2011.

### 2.3. Participants

Eligible participants were identified and recruited through schools, physiotherapy services, and public clinics in the Auckland region of New Zealand. Eligible children were aged between 5 and 18 years and diagnosed with CP GMFCS level II. Potential participants were excluded if unable to stand on the vibration plate or understand and follow the researcher's instructions for the assessment and intervention protocol. Additional exclusion criteria were a recent fracture (within the last eight weeks), acute thrombosis, muscle/tendon inflammation, nephrolithiasis, discopathy, arthritis, as well as any use of anabolic agents, glucocorticoids (other than asthma inhalers), bisphosphonates, or growth hormone. No participants received botulinum toxin injection during the study or in the three months preceding its commencement. Note that we asked participants not to start on any new forms of rehabilitation during the study. However, participants were advised to continue with any regular activities or other forms of physiotherapy they were already involved with prior to their enrolment into our study.

### 2.4. Intervention protocol

sVT was performed on the Galileo Basic vibration plate (Novotec Medical, Pforzheim) four times a week for 20 weeks, following a specific intervention protocol (17). Participants began with five 1 min sessions of vibration therapy at a frequency of 12 Hz. The frequency and duration of the sessions were gradually increased over time, in accordance with the training protocols of previous studies (17). From week 5 (21st session), all participants trained for 15 min at a frequency of 20 Hz with an amplitude of 2 mm, four times a week. The 15 min protocol consisted of five 3 min bouts of sVT separated by 3 min breaks (17).

During sVT, participants stood barefoot on the vibration plate with feet apart and parallel, and knees slightly bent. Sessions took place either at school or at home, depending on the needs of each family. Once a week, an experienced physiotherapist supervised the sVT sessions at school or at home to monitor the participants' progress, and to ensure the training procedures were carried out correctly. The researchers were also available to provide additional feedback/support to the parents/caregivers supervising the home sessions.

### 2.5. Measures

All clinical assessments were conducted at the Maurice and Agnes Paykel Clinical Research Unit (Liggins Institute, University of Auckland) by an experienced exercise physiologist and physiotherapist. At the beginning of each assessment, the participants' anthropometric data (i.e., height and weight) were measured and their body mass index (BMI) calculated. Height, weight, and BMI were subsequently transformed into age- and sex-adjusted *z*-scores as per World Health Organization standards (18).

### 2.6. Study outcomes

To ascertain the feasibility of the 15 min vibration therapy protocol, the key objectives were to assess: (1) adherence to the sVT protocol; and (2) adverse events.

Adherence was defined as the percentage of sessions completed out of the 80 prescribed sessions, where a sVT session was deemed to be complete if the participant performed all five sVT sets. Data on the number of sessions were extracted from the sVT diary that the participant and their family or school physiotherapist kept during the study (17). The diary was also used to record adverse events, if any (17).

Adverse events were categorized as either transient or serious. Transient adverse events were defined as those that are temporary and resolved within minutes after the sVT session completion, did not require medical intervention, and did not interfere with the children's ability to follow the treatment protocol (e.g., skin redness, itching, or warmth, and muscle soreness). Serious adverse events were defined as outcomes likely to be caused by sVT and leading to its discontinuation, such as any long-lasting pain, dizziness, impaired balance, hypotension, or hypoglycemia.

The criteria to ascertain the feasibility of the proposed 15 min sVT protocol were:

(1) Mean adherence ≥75%; AND
(2) The absence of any reported serious adverse events.

Secondary outcomes were examined in exploratory analyses on the potential effectiveness of the 15 min sVT protocol on body composition and physical function. The main functional parameter of interest was mobility, given improving walking ability is one of the main rehabilitation goals for patients with CP (19). Mobility was measured by the 6 min walk test (6MWT), where the participants is asked to walk as fast as possible for exactly 6 min, with the total distance covered recorded as the outcome of interest (20). Body composition was assessed using whole-body dual-energy x-ray absorptiometry (DXA) scans (Lunar Prodigy 2000, General Electric, Madison, WI, USA). The main parameters of interest were total body (TB) BMD and bone mineral content (BMC), the android-to-gynoid-fat ratio (a marker of abdominal adiposity), fat mass, and lean (muscle) mass. Physical function was assessed with the Leonardo Mechanography® Ground Reaction Force Platform (GRFP) (Novotec Medical, Pforzheim, Germany). The GRFP assessments included the chair-rising test (CRT) and the single two-leg jump test (STLJT), reported as the average rise time (e.g., mean time per repetition for repetitions 2 to 4) in seconds and the jump height in centimeters, respectively (21). During the CRT, participants stood up and sat down five times as fast as possible on a specially designed seat placed on the GRFP. The STLJT consisted of jumping as high as possible with both legs and landing on the forefoot (21). Both assessments were performed three times, with the best of three results used for analyses.

### 2.7. Sample size calculation

Based on an observed standard deviation of 46.5 m or 10.9% for the change from baseline in the 6MWT distance covered among 31 youth with CP GMFCS II (10), a study with 15 participants would be powered to detect a statistically significant difference from baseline of 10% after sVT, with 90% power and α = 0.05.

### 2.8. Data analyses

The distance covered in the 6MWT after the 20-week intervention was compared to baseline using a general linear mixed model, including assessment (pre-sVT and post-sVT) as the main predictor, adjusting for the participant's age and including participant ID as a random factor. The same model structure was adopted for secondary outcomes. Potential differences between pre- and post-sVT are reported as the adjusted mean differences and respective 95% confidence intervals.

Further, the time taken to achieve the distance milestones in the 6MWT was also compared using linear mixed models based on repeated measures constructed as above, but including milestone (50, 100, 150, 200, 250, 300, 350, 400, 450, and 500 m) and an interaction term (assessment*milestone) as independent variables. In addition, the *p*-values for the pre- vs. post-sVT comparisons at each milestone were adjusted for multiple comparisons using the Holm–Šídák sequential procedure (22). Lastly, the potential change in the proportion of participants reaching the 400 m mark in the 6MWT was assessed using a McNemar's test for paired data.

All statistical tests were two-tailed, and, except for the 6MWT milestones, the significance level was maintained at 5%. Statistical analyses were carried out in SAS v.9.4 (SAS Institute, Cary, NC, USA).

## 3. Results

Fifteen participants aged 5.2–17.4 years were recruited, including 6 females and 9 males, all of NZ European ethnicity (Table 1). Of the 15 participants, 8 children (57%) were diagnosed with global developmental delay and 4 (23%) had medically well-controlled seizures.

**Table 1:** Baseline characteristics of study participants.

*n*		15
Age (years)		12.4 [9.9, 15.7]
Gender	Female	6 (40%)
	Male	9 (60%)
Height (cm)		148.8 ± 4.5
Weight (kg)		42.4 ± 4.7
BMI (kg/m^2^)		18.9 ± 1.2
Type of CP	Spastic	8 (53%)
	Ataxic	3 (20%)
	Hypotonic	1 (7%)
	Mixed	1 (7%)
	Not specified	2 (13%)

BMI, body mass index, CP, cerebral palsy.

Gender and type of CP data are *n* (%); age data are the median [quartile 1, quartile 3]; other continuous data are means ± standard deviations.

Mean adherence to the 15 min sVT protocol was relatively high at 83% [quartile 1 = 63.9%; median = 84.7%; quartile 3 = 97.2%). The main reported reason for missing individual sVT sessions was a lack of time. All recruited participants completed the prescribed 20 weeks of sVT, without any study dropouts. Importantly, there were no transient or serious adverse events reported.

**Figure 1:**
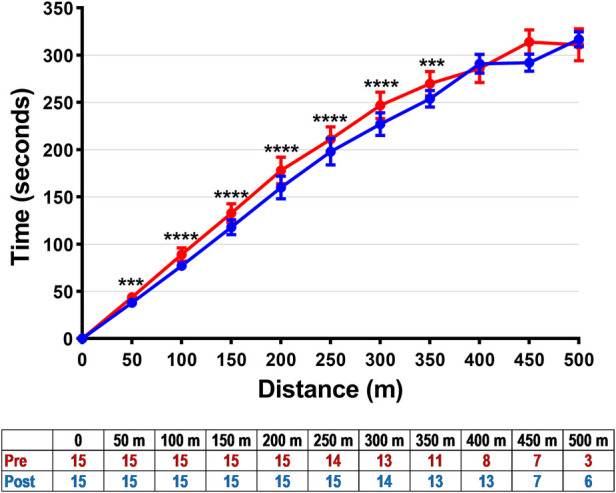
Distance milestones in the 6 min walk test and the time taken to reach them at baseline (red) and after 20 weeks of side-alternating vibration therapy (blue) among children and adolescents with cerebral palsy. Data are means ± standard errors. ***p < 0.001 and ****p < 0.0001 for the differences between pre- and post-sVT at a given time point, after a Holm–Šídák correction for multiple comparisons, derived from a linear mixed model based on repeated measures. The table shows the number of participants who reached a given milestone.

After 20 weeks of sVT, participants appeared to have increased the distance covered in the 6MWT by 43 m on average (+10%; *p* = 0.0018) (Table 2). In addition, following sVT, participants reached most of the individual 6MWT milestones earlier (Figure 1). There was also some evidence that more participants were able to reach the furthest milestones, so that 13 participants (87%) managed to reach the 400 m mark after sVT compared to 8 (53%) at baseline (+33%; *p* = 0.063) (Figure 1).

**Table 2:** Mobile and functional outcomes among children and adolescents with cerebral palsy at baseline and after 20 weeks of side-alternating vibration therapy.

Assessment	Parameter	*n*	Baseline	Post-intervention	aMD
6MWT	Distance (m)	15	428 ± 112	472 ± 101	**43** (19, 68)*
CRT	Av.rise time (seconds)	10	0.94 ± 0.32	0.91 ± 0.29	−0.07 (−0.25, 0.11)
STLJT	Jump height (cm)	13	19.2 ± 8.4	21.5 ± 7.3	2.7 (−1.6, 6.9)

aMD, adjusted mean differences; Av.rise time, average rise time; 6MWT, 6 min walk test; CRT, chair-rising test; STLJT, two-leg single-jump test.

Baseline and post-intervention data are means ± standard deviations; aMD are means and 95% confidence intervals, adjusted for participant’s age.

* *p* = 0.001 for the difference between baseline and post-training.

Statistically significant differences (at *p*<0.05) are shown in bold.

There were no observed changes in body composition apart from a small increase in spine BMD (+5%; *p* = 0.013), even though spine BMD *z*-score was unchanged after the intervention (Table 3). There were no observed differences in the GRFP assessments, namely the CRT or the STLJT (Table 2).

**Table 3:** Body composition of children and adolescents with cerebral palsy at baseline and after 20 weeks of side-alternating vibration therapy.

Assessment	Parameters	Baseline	Post-training	aMD
Anthropometry	*n*	15	15	
** **	Weight *z*-score	−0.18 ± 1.89	−0.20 ± 1.83	−0.03 (−0.25, 0.19)
** **	Height *z*-score	−0.22 ± 1.45	−0.28 ± 1.47	−0.06 (−0.22, 0.10)
** **	BMI *z*-score	−0.04 ± 0.43	0.03 ± 0.47	0.07 (−0.27, 0.40)
Body composition	*n*	15	15	
** **	A/G ratio	0.717 ± 0.211	0.743 ± 0.190	0.036 (−0.010, 0.083)
** **	Total fat mass (kg)	10.85 ± 8.53	11.82 ± 9.31	0.77 (−0.14, 1.67)
** **	Total lean mass (kg)	31.00 ± 11.20	31.57 ± 11.37	−0.42 (−1.21, 0.37)
** **	Total BMD (g/cm^2^)	0.947 ± 0.129	0.957 ± 0.134	0.001 (−0.014, 0.016)
** **	Total BMD *z*-score	−0.66 ± 1.73	−0.72 ± 1.66	0.04 (−0.30, 0.37)
** **	Leg BMD (g/cm^2^)	0.940 ± 0.199	0.942 ± 0.201	−0.013 (0.039, 0.013)
** **	Spine BMD (g/cm^2^)	0.802 ± 0.167	0.838 ± 0.204	**0.032** (0.008, 0.057)*
** **	Spine BMD *z*-score	−0.51 ± 1.79	−0.69 ± 1.78	−0.06 (−0.39; 0.27)
** **	Total BMC (g)	1,625 ± 683	1,701 ± 744	24 (−29, 77)
** **	Leg BMC (g)	540 ± 240	555 ± 244	−6 (−26, 13)
** **	Spine BMC (g)	149 ± 79	159 ± 94	7 (−7, 21)

A/G ratio, android-to-gynoid-fat ratio; aMD, adjusted mean difference; BMC, bone mineral content; BMD, bone mineral density; BMI, body mass index.

Baseline and post-intervention data are means ± standard deviations; aMD are means and 95% CI adjusted for participant's age.

* *p* = 0.012 for the difference between baseline and post-intervention.

Statistically significant differences (at *p*<0.05) are shown in bold.

## 4. Discussion

This study showed the 15 min 20-week sVT protocol is feasible in children and adolescents with CP GMFSC level II. Adherence was high (83% on average), and sVT was well-tolerated with no adverse events reported, and no dropout of participants so that all completed the 20-week intervention period. In addition, the obtained data indicated a likely improvement on mobility after sVT as shown in the 6MWT.

It is important to note that the additional training time (from 9 to 15 min per session) did not adversely affect adherence to the sVT protocol. On average, 83% of prescribed sVT sessions were completed by the study's participants, which is similar to the adherence levels of 77%–85% in previous studies using a 9 min sVT protocol in participants with CP and other musculoskeletal disorders (13, 23, 24).

Our preliminary results suggest a notable 10% improvement in the distance covered by the 6MWT, which is consistent with the results from other studies using variations of the 9 min protocol in children with CP (10, 13) and other musculoskeletal conditions (24). Gusso et al. (10) reported an 11% improvement in the distance covered in the 6MWT among 34 children and adolescents with CP GMFCS level II after 20 weeks of sVT (10), while Vesey et al. (24) reported a 9% improvement among seventeen children and adolescents with musculoskeletal disorders using the same sVT protocol (24). Another large study showed that the improvement in the 6MWT distance after sVT was greater in children with higher degrees of physical impairment (i.e., GMFCS levels III and IV) in comparison to participants with GMFCS level II (23). The specific mechanisms underpinning the beneficial effects of sVT on walking capacity in children and adolescents with CP are not well described. However, proposed mechanisms include the activation of alpha motor neurons (7) and Golgi tendon organs (25), facilitation of the sensory input (26), proprioception stimulation, and the secretion of hormones such as growth hormone and testosterone (7, 27). Further, sVT can stimulate spinal and supraspinal functions, leading to improved nervous control of muscle fiber recruitment (27). These mechanisms may allow for greater activation of the musculoskeletal system in individuals with limited ability to perform weight-bearing activities, providing a potential pathway for increasing mobility (i.e., walking capacity). Irrespective of the underlying mechanisms, improving walking capacity is an important therapeutic goal for children with CP (19), particularly since such improvement could lead to increased participation in other forms of physical activity. By improving activity levels through to adulthood, there could be overall health benefits, such as improvements in bone and muscle function. Thus, the inclusion of sVT in the health management of young people with mild CP could lead to substantial improvements in quality of life in the long term through increased functional mobility.

The tested 15 min sVT protocol also suggested an increase in spine BMD but not the corresponding *z*-score. Studies on this outcome have described conflicting findings, and a meta-analysis investigating sVT in children with CP reported improvements in femur BMD but not in lumbar spine BMD when compared with controls (28). Further research is needed to reliably ascertain the impact of sVT and protocol length on BMD in young people with CP. Importantly, natural development and growth and its effect on bone density in children and adolescents should be considered while analyzing the study results (29, 30), but our statistical models did adjust for the participant's increase in age over the study period. In addition, sVT interventions for a minimum of 6 months have been suggested as necessary to achieve improvements in bone health (31), which might not be detectable in shorter sVT protocols. While our findings suggest that the 15 min sVT protocol produced similar outcomes to the 9 min protocol used in previous studies (10, 23), it remains unclear whether longer sessions could result in greater improvements to health outcomes.

As a feasibility study, its design had limitations. We only recruited participants with CP of GMFCS level II, so that our findings cannot be extrapolated to children with more severe forms of CP or other disorders. Our exploratory analyses on health outcomes did not account for other concurrent forms of rehabilitation, but no participants started on new forms of rehabilitation during the study.

Nonetheless, our primary focus was to assess the safety and acceptability of a 15 min sVT protocol, and, to our knowledge, this study was the first to show that longer sVT sessions are feasible for children and adolescents with CP. Although our study was not specifically designed to evaluate the efficacy of sVT on physical function and body composition, our preliminary findings suggest potential health benefits associated with a 15 min sVT protocol in this population. These findings warrant further investigation through gold standard randomized controlled trials. In addition, it would be valuable to compare the effects of different sVT protocol lengths, such as 9 min vs. 15 min. Such trials should also investigate the potential effects of sVT on muscle metabolism and other relevant biochemical markers, including growth factors and on sensory deficits seen in children with CP.

## Data Availability

The datasets presented in this article are not readily available because Ethics approval has not been granted to share the dataset. Requests to access the datasets should be directed to Dr Silmara Gusso s.gusso.@auckland.ac.nz.
